# Predictive factors of diagnostic and therapeutic divergence in a nationwide cohort of patients seeking second medical opinion

**DOI:** 10.1186/s12913-021-06936-w

**Published:** 2021-09-01

**Authors:** Stéphane Sanchez, Isabelle Adamowicz, Jan Chrusciel, Philippe Denormandie, Pierre Denys, Laurent Degos

**Affiliations:** 1 Public Health and Performance Department, Champagne Sud Hospitals, 101 avenue Anatole France, 10000 Troyes, France; 2 Scientific Department, Deuxième Avis, Paris, France; 3 Orthopedic Department, Raimond Poincaré Hospital, APHP, Garches, France; 4 Urology and Neurology Rehabilitation Department, Raimond Poincaré Hospital, APHP, Garches, France; 5 French academy of sciences, Paris, France

## Abstract

**Objectives:**

The aim of this study was to describe the profile of patients who sought a second medical opinion (SMO) on their therapeutic or diagnostic strategy using nationwide data from a French physician network dedicated to SMOs.

**Methods:**

An observational cohort study was conducted and the study population consisted of patients residing in France or in the French overseas territories who submitted a request for an SMO through a dedicated platform between January 2016 and October 2020. Patient characteristics were compared between convergent and divergent SMOs. The divergent rate for all patients excluding those with mental diseases were described. Logistic regression was used to estimate the probability of a divergent SMO according to patient characteristics.

**Results and discussion:**

In total, 1,552 adult patients over several French regions were included. The divergence rate was 32.3 % (*n* = 502 patients). Gynecological [Odds Ratio (OR) and 95 % CI: 5.176 (3.154 to 8.494)], urological [OR 4.246 (2.053 to 8.782)] and respiratory diseases [OR 3.639 (1.357 to 9.758)] had the highest probability of a divergent SMO. Complex cases were also associated with a significantly higher risk of a divergent opinion [OR 2.78 (2.16 to 3.59)]. Age, sex, region and profession were not found to be predictive of a divergent second opinion.

**Conclusions:**

Policymakers should encourage new research on patient outcomes such as mortality and hospitalization rates after a SMO. When proven effective, SMO networks will have the potential to benefit from specific public funding or even play a key role in national healthcare quality improvement programs.

**Supplementary Information:**

The online version contains supplementary material available at 10.1186/s12913-021-06936-w.

## Introduction

An expert second medical opinion (SMO) enables a patient to confirm or re-evaluate a diagnosis and/or a treatment recommended by a general practitioner (GP) or specialist. The practice of seeking an SMO has been described in literature for a range of disease types [1–12], with different approaches according to the source of the referral given (patient or doctor), level of specialization of the physician performing the SMO (general practitioner or another specialist) and the modality of the second analysis (consultation or history and chart review) [3, 9]. In the United States, SMOs were first known as a way to control rising healthcare costs, for example by preventing unnecessary elective surgery and for this purpose SMOs were mandatory for Medicaid recipients in the 1980 s but later appeared as a way to improve healthcare quality [13].

SMO can result in a change of diagnosis, treatment or prognosis in 10–62 % of cases [6]. This wide variation may be due to healthcare provider divergence, variations in the quality of the methodology used in the studies, or having small sample sizes.

A German study, where the cost of SMOs were stated to be paid either by insurance companies or the patient, found that 64.8 % of the initial therapeutic strategies were not confirmed in the second opinion consultations. In this study, SMOs were sought primarily for orthopedic conditions (knee, spine, hip and shoulder) with an agreement rate as low as 26.2 % for the most common family of conditions in the program (diseases of the knee) [14].

In Israel, SMOs are funded either through the universal National Health Insurance, voluntary insurances programs or out-of-pocket payments. A cross-sectional study conducted on a representative random sample of the general adult population reported that 56 % of 305 SMO seekers considered that there was a difference in diagnosis or treatment between the first opinion and the second consultations. Ophthalmologic and gynecological motives were also frequent in this study (9 and 8.1 % respectively, after orthopedics which accounted for 32.3 % of the sample) [15].

In another population-based study conducted in Israel, the type of insurance did not influence divergence rates for most specialties, with the exception of a notably higher divergence rate for neurological disease in patients who asked for a second opinion with their supplementary insurance (9 % vs. 3 %) [16].

Cancer patients are well represented among second opinion seekers, and there is a high potential for changes to the initial therapeutic strategy. Breast cancer, has been associated with a wide use of SMOs, especially at the early stage [4, 8, 10]. For example, in a breast cancer consultation study, 43 % of patients had a change of diagnosis and 23 % had an additional tumor found by a SMO [4]. Although self-referral to a second physician could be associated with higher sensitivity in cancer detection [10], targeted SMO referral strategies in breast cancer cases may have a more favorable profile in cost-benefit analyses [8]. The potential for therapeutic change is also clear for less frequent cancers. A study conducted in a reference cancer treatment center in the US recruited two surgeons to review second opinion radiological assessments of pancreatic ductal adenocarcinoma. Upon seeing the second opinion evaluation, they proposed a change of the patients’ management recommendation in 38,4 and 20.0 % of cases respectively [17].

Higher education status, socio-economic status and female gender [5] have, to date, been associated to the characteristics of patients that seek an SMO during diagnosis [5, 9].

Understanding which class of diseases may require an SMO to improve healthcare quality could help with the efficient care planning as patients who have divergent SMO also show higher healthcare expenses [3]. The class of diseases where the percentage of divergence is higher could therefore be a target for quality improvement interventions. The evidence on situations where the benefit of an SMO outweighs the cost of an additional consultation is, however, still limited [18–20]. The aim of this study was to describe the profile of patients who sought a SMO on their therapeutic or diagnostic strategy using nationwide data from a French physician network dedicated to SMOs.

## Methods

### Study population

A prospective observational cohort study was conducted using nationwide data from a French SMO platform (https://www.deuxiemeavis.fr/). Patients were included if they resided in France or an overseas French territory and submitted a request for an SMO through the platform between 1 and 2016 and 1 October 2020. All participants consented to the use of their data for research purposes. Mental health disorders were not subject to a SMO in accordance with the existing literature [12].

### SMO platform process

Patients accessed the SMO platform online via a secure connection after creating a free personal account. Patients were then asked their category of disease and their choice from a list of SMO experts. They completed a detailed medical questionnaire related to their concerns and were encouraged to ask the medical inquiries they wanted to obtain an SMO for. Patients could add relevant medical examinations to their request. The *Second Look* network has 220 experts in various French public and private hospitals and healthcare centers. These experts were selected according to their academic position, clinical experience, international activities and approval by a scientific jury. The SMO platform also partnered with a variety of private insurers to obtain consultations at competitive prices for its patients.

A designated expert was then notified and could either accept or decline the request. If the request was accepted by the expert, a medical analysis of the request was performed. A secure messaging system was available in case additional information was required by the expert. The expert completed a written report which was then shared with the patient. The report stated if the SMO was convergent or divergent. The expert was also asked to rank the level of complexity of the SMO. The patient had the possibility to message the expert back in case of additional questions.

The SMO report was also sent to the patients’ general practitioner (GP) with the patient’s consent. The patients were also asked to rate their satisfaction with the SMO platform at case completion.

### Data collection

The following variables were recorded for all case submitted: disease, class of diseases, expert name, level of complexity (simple or complex), divergent or convergence of the SMO, and time duration of the review. For each patient, the age, sex, region of residence, employment category, and whether the GP was aware that the patient was seeking an SMO or not, were collected.

The dependent variable used for statistical modelling was convergence or divergence of both diagnosis and treatment between the first and second opinion. For each case, if the diagnosis and treatment were fully consistent between the first and second opinion, the dependent variable was coded as convergent. In all other cases (diagnostic and/or therapeutic divergence) the dependent variable was coded as divergent.

The dependent variable was designed as a binary variable and directly coded as such during data collection. No quantitative divergence scale was used, therefore there was no need to determine a specific threshold to use for the study.

### Statistical analysis

Quantitative variables were presented as median and 1st and 3rd quartiles (Q1, Q3) if non-normally distributed, and qualitative variables were presented as number (%). We compared patient characteristics between convergent and divergent SMO. Groups were compared using the chi square or Fisher’s exact test as appropriate, and the Mann-Whitney U test was used to compare the time required to prepare the SMO. A planned exploratory analysis was performed to compare simple and complex cases.

Logistic regression was used to estimate the probability of a divergent SMO according to patient characteristics. Missing data were handled by multiple imputation with m = 100 imputations. All analyses were performed using R version 4.0.2 (www.R-project.org). Multiple imputation was performed with the *mice* package (Stefan Van Buuren).

### Ethical considerations

The database used for this study was fully anonymous. The SMO platform received special authorization from the French national commission for data privacy (*Commission Nationale Informatique et Libertés*, CNIL) when it was launched (at the beginning of 2016), in compliance with a legislative requirement that has since been lifted. Patients provided informed consent that their anonymous data may be used for research and quality improvement purposes. No additional approvals were required according to the French legislation. According to the ethical rules of the SMO platform scientific council, an SMO expert was not allowed to be directly in charge of the patient.

## Results

A total of 1,552 patients were included in the study. The divergence rate was 32.3 % (502/1552 patients). The main characteristics of the study population are shown in Table 1. In the univariate analysis, case complexity and age were associated with a divergent SMO. There was no difference between groups for the region of residence or employment category.

**Table 1:** Patient characteristics according to divergence of the second look opinion

Characteristics	Convergent	Divergent	Missing n or %	***P***-value	French population (% of category in general population)
	***N=1050***	***N=502***	***N=384***		***N = 67,287,241***
Male Sex	420 (40.1%)	178 (35.5%)	0.2%	0.08	32,397,179 (48.8%)
Specific or exceptional complexity	208 (21.1%)	193 (39.7%)	23.7%	<0.0001	
Satisfaction with the service provided					
* Quite satisfied*	27 ( 3.8%)	15 (3.9%)	29.9%	0.67	
* Not at all satisfied*	4 ( 0.6%)	2 (0.5%)			
* Satisfied*	151 (21.1%)	69 (18.2%)			
* Very satisfied*	532 (74.5%)	294 (77.4%)			
Expert recommendation by patient	675 (97.0%)	365 (97.1%)	31.7%	0.99	
Choice made by the patient	722 (68.8%)	359 (71.5%)	-	0.27	
Median time required for opinion, days [IQR]	2.00 [0.47, 5.16]	2.28 [0.66, 5.20]	0.3%	0.09	
Region of residence					
* Ile de France*	295 (30.1%)	143 (30.4%)	8.5%	0.133	12,291,557 (18.3%)
* Auvergne Rhone Alpes Bourgogne Franche Comte*	119 (12.1%)	54 (11.5%)			10,858,663 (16.1%)
* Bretagne Normandie Pays de la Loire Centre Val de Loire*	123 (12.7%)	82 (17.4%)			13,056,103 (19.4%)
* Hauts de France Grand Est*	208 (21.2%)	83 (17.7%)			11,523,797 (17.1%)
* Nouvelle Aquitaine*	77 (7.8%)	30 (6.4%)			6,018,424 (8.9%)
* PACA, Occitanie*	145 (14.8%)	74 (15.7%)			11,029,432 (16.4%)
* Corsica*^a^	5 (0.5%)	0 (0.0%)			345,867 (0.5%)
* Overseas territories*^a^	9 (0.9%)	4 (0.9%)			2,163,398 (3.2%)
Age groups					
* Teenagers<18 years*	50 ( 4.8%)	21 (4.2%)	-	<0.0001	15,252,608 (22.7%)
* Adults aged 36 to 50 years*	308 (29.3%)	136 (27.1%)			12,887,561 (19.2%)
* Adults aged 19 to 35 years*	239 (22.8%)	181 (36.1%)			13,177,128 (19.6%)
* Adults aged 51 to 65 years*	256 (24.4%)	97 (19.3%)			12,790,894 (19.1%)
* Older adults aged 66 to 80 years*	183 (17.4%)	62 (12.4%)			9,206,549 (13.7%)
* Older adults aged 81 to 95 years*	14 (1.3%)	5 (1.0%)			3,515,308 (5.2%)
Professional status					
* Intermediate profession*	248 (33.0%)	148 (38.4%)	30.6%	0.07	14,041,617 (20.9%)
* Self-employed, business owner or manager*	230 (30.6%)	95 (24.7%)			7,373,204 (11.0%)
* Other*	67 (8.9%)	39 (10.1%)			487,932 (0.7%)
* Currently unemployed (job seekers or students)*	57 (7.6%)	39 (10.1%)			23,912,440 (35.5%)
* Manal worker*	25 (3.3%)	11 (2.9%)			5,204,615 (7.7%)
* Retired*	124 (16.5%)	53 (13.8%)			16,267,433 (24.2%)

^a^Region not included in multivariable analysis due to insufficient sample size

A summary of divergence rates is presented in Table 2 according to broad disease categories. Overall, the type of disease was significantly related to the rate of divergence (*p* < .001). Diseases with four or more diverging opinions are described in Table 3.

**Table 2:** Divergence rate by family of diseases

Group	Convergent (n)	Divergent (n)	Proportion divergent (%)	Missing (n)
	***N = 1050***	*** N = 502***	-	***N = 384***
Orthopedics and rhumatological diseases	408	134	24,7	131
Cardiovascular diseases	45	29	39,2	18
Reproductive diseases	62	25	28,7	21
Nervous system and sensory organ diseases	106	41	27,9	27
Skin disorders	17	6	26,1	6
Digestive system diseases	33	16	32,7	13
Tumours, cancer, hematological diseases	187	48	20,4	94
Endocrine and metabolic diseases	38	20	34,5	13
Gynecological diseases	121	150	55,4	30
Respiratory diseases	10	9	47,4	4
Urological diseases	21	22	51,2	27
Other	2	2	50,0	0

**Table 3:** Class of diseases where at least four cases had divergent second opinions

Class of Diseases	Convergent (n)	Divergent (n)	Divergence rate (%)
Orthopedics and rhumatological diseases			
* Herniated lumbar disc*	51	13	20.3 %
* Degenerative disc disease*	31	10	24.4 %
* Lumbago (chronic lumbar pain)*	12	10	45.5 %
* Lumbar arthritis*	7	6	46.2 %
* Ankle impingement (bone or tissue)*	5	5	50.0 %
* Cervical arthritis*	3	4	57.1 %
* Fracture of the humerus (consequences included)*	7	4	36.4 %
* Fracture of the wrist or forearm (consequences included)*	6	4	40.0 %
* Meniscal lesions*	15	4	21.1 %
Cardiovascular diseases			
* Coronary artery disease*	4	8	66.7 %
Reproductive diseases			
* Female infertility*	51	16	23.9 %
* Azoospermia*	4	4	50.0 %
Nervous system and sensory organ diseases			
* Adult epilepsy*	4	6	60.0 %
* Herniated cervical disc*	18	5	21.7 %
* Multiple sclerosis*	6	5	45.5 %
Digestive system diseases			
* Crohn’s disease*	3	5	62.5 %
Tumour, cancer, hematological diseases			
* Prostate cancer*	30	16	34.8 %
* Bladder cancer*	8	4	33.3 %
Endocrine and metabolic diseases			
* Goitre or thyroid nodules*	7	5	41.7 %
* Hyperthyroidism*	10	5	33.3 %
Gynecological diseases			
* Endometriosis*	90	137	60.4 %
* Uterine fibroma*	12	11	47.8 %
Respiratory diseases			
* Chronic obstructive pulmonary disease*	3	4	57.1 %
Urological diseases			
* Prostate adenoma*	14	14	50.0 %
Total	401	305	43.3 %

The overall divergence rate for orthopedic diseases was 24.7 %. Prostate adenoma had a divergence rate of 50 % (*n* = 14/28 patients). Regarding gynecological diseases, endometriosis and uterine fibroma had high divergence rates of 60.4 and 47.8 % respectively. In the cancer group, prostate and bladder cancers often showed a divergent second opinion with divergence rates of 34.8 % (*n* = 16/46) and 33.3 % (*n* = 4/12) respectively.

Among cardiovascular diseases, the divergence rate for coronary artery disease was 66.7 %. In the category of endocrine and metabolic diseases, there were five divergent cases for goiter/thyroid nodules and hyperthyroidism (41.7 and 33.3 % divergence rate respectively), while chronic obstructive pulmonary disease had the most (absolute) divergent cases among the respiratory diseases (4/7 divergent cases, 57.1 %). The full set of results with the list of all diseases is presented in Additional file 1.

The factors associated with a divergent SMO by multivariate analysis are shown in Table 4. Cardiovascular, endocrine/metabolic, gynecological and respiratory diseases were associated with a significantly higher risk of having a divergent opinion (Table 4). Complex cases were also associated with a significantly higher risk of a divergent opinion (Odds ratio (OR) 2.78;95 % CI, 2.15 to 3.59). Furthermore, the time required to produce an SMO report was significantly longer in complex cases. Model performance was acceptable: C- Statistic AUC 0.706 (0.694 to 0.719) and Nagelkerke pseudo-R2 0.16 (0.15 to 0.18). These findings are presented in Additional file 2.

**Table 4:** Factors associated with a divergent second medical opinion by multivariate logistic regression analysis

Characteristics	OR	95 % CI		*P*-value
Class of diseases				
* Oncology and hematological diseases*	1 (Ref)	-	-	
* Cardiovascular diseases*	2.609	1.441	4.723	< 0.0001
* Reproductive diseases*	1.746	0.923	3.303	
* Skin disorders*	1.356	0.483	3.808	
* Digestive system diseases*	1.759	0.867	3.570	
* Endocrine and metabolic diseases*	2.072	1.089	3.944	
* Gynecological diseases*	5.176	3.154	8.494	
* Respiratory diseases*	3.639	1.357	9.758	
* Orthopedics and rhumatological diseases*	1.266	0.860	1.864	
* Nervous system and sensory organ diseases*	1.267	0.761	2.11	
* Urological diseases*	4.246	2.053	8.782	
Complexity				
* Normal or common*	*1 (Ref)*	*-*	*-*	*< 0.0001*
* Specific/exceptional*	*2.784*	*2.157*	*3.592*	
Time required for file review	0.985	0.959	1.011	0.26
Region of residence				
* Ile-de-France*	1 (Ref)	-	-	0.18
* Auvergne-Rhône-Alpes, Bourgogne-Franche-Comte*	1.044	0.680	1.601	
* Bretagne, Normandie, Pays de la Loire, Centre-Val de Loire*	1.64	1.121	2.399	
* Hauts-de-France, Grand Est*	1.125	0.784	1.613	
* Nouvelle-Aquitaine*	0.98	0.583	1.647	
* PACA, Occitanie*	1.209	0.817	1.79	
Professional status				
* Intermediate profession*	1 (Ref)	-	-	0.39
* Self-employed, business owner or manager*	0.78	0.563	1.082	
* Other*	0.915	0.557	1.502	
* Currently unemployed (job seekers or students)*	0.813	0.499	1.323	
* Manual labourer*	0.944	0.449	1.983	
* Retired*	1.291	0.743	2.244	
Age category				
* Adult (36 to 50 years)*	1 (Ref)	-	-	0.40
* Adult (19 to 35 years)*	1.219	0.892	1.666	
* Adult (51 to 65 years)*	0.949	0.658	1.368	
* Child (< 18 years)*	1.211	0.654	2.24	
* Older adult (66 to 80 years)*	0.705	0.410	1.211	
* Older adult (81 to 95 years)*	0.573	0.185	1.771	
* Sex Male (Ref = Female)*	1.25	0.955	1.637	0.10

## Discussion

In this nationwide study with data on 1,552 SMO requests, we found that gynecological, respiratory, endocrine/metabolic and cardiovascular diseases were more likely to have a divergent SMO. The complexity of the case was also found to be associated with a higher likelihood of a divergent SMO.

In the literature, the main reasons proposed for seeking a SMO were persisting symptoms, absence of diagnosis or the need for confirmation of a diagnosis, the need for more information, questioning the need for surgery [1] (such as total mastectomy), and a desire to change the proposed treatment [6, 7]. In some cases, these reasons are largely independent of the quality of patient-physician relationship, although this could be less frequent in patients with a low education level [21]. Some studies have shown a link between the level of education and the tendency to seek an SMO [9]. Although our study did not record the level of education of patients directly, we found no relation between the patient’s employment and the divergent rate in the SMO, considering the professional status as a proxy for education level. There was no evidence in our study that those with higher-level jobs requested a SMO more frequently. Other factors previously reported to be associated with the propensity to request a SMO (such as age, sex, socio-economic category, immigrant status and income) were also not found in our study [16].

The divergence rates observed in our study were similar to the literature on SMOs [6]. The divergent rate for orthopedics in our study (24.7 %) was similar to the rate reported by Chalian et al., which had divergences of categories 4 and 5 (“likely to change patient management”) in 26.2 % of musculoskeletal radiological examinations [22]. However, numerous orthopedic complaints (such as low back pain with 45.5 % divergence in our study) were influenced by psychological factors and could fall into the “functional complaint” category, a known source of why SMOs are requested [23].

Regarding endocrine diseases, our findings were also similar to other literature findings, with a 34.5 % divergence rate compared to 28.6 % reported in the specific context of thyroid cytology [24]. The thyroid is known as a high-risk area for misdiagnosis [25], which is consistent with endocrine diseases being associated with therapeutic divergence. Other head and neck region cancers also showed a high percentage of divergent diagnoses [26] (such as salivary glands cancer 2/3 divergent diagnoses in our study, 66.7 %), although the study sample was small in this subgroup.

For neurological diseases, there was a divergent opinion in 27.9 % of cases in our study, which compared favorably with data from the literature reporting divergent SMO in 59.8 % of patients initially recommended for spine surgery [27]. The divergence rate for urological malignancies (approximately one third of divergence for prostate and bladder) was higher in our study than in the literature [28].

In a study of 286 referrals, Van Such et al. found that the final diagnosis (after review) was better defined in 66 % of cases and differed in 21 % of cases [3]. Other authors reported divergences in 44 % of breast cancer cases and various other ranges likely due to insufficient sample sizes [4, 8, 10]. Some authors reported lower rates of agreement, but mainly in diseases with a predominantly symptomatic presentation such as unspecific pain that may be hard to diagnose and/or treat [5].

Two limitations should be mentioned about the similarity of our results to literature. In our study, the first and second opinions could not be considered as independent. A prior study showed that knowledge of a previous decision can influence second opinion therapeutic strategy [29]. Moreover, in some of the available studies, the second opinion was obtained several days or weeks after the first consultation and the patient’s status could have changed between the first and second opinions.

The need for more information has been associated with physician distrust [30], and some patients expected more personalized communication during an SMO consultation [31]. As patients are expected to feel better informed after an SMO, it has been argued that the possibility of this re-examination is part of the new patient-centric medical paradigm [32, 33]. It should be noted that patients increasingly rely on written material available online to get a SMO regarding their diagnosis rather than on health professionals [34, 35].

Lastly, the use of social media (including pages dedicated to healthcare professionals) to obtain a SMO is increasingly frequent and raises ethical concerns with respect to the physicians’ responsability [36]. Despite evident limitations, platforms like WhatsApp allow patients or physicians to rapidly obtain help, for example by requesting expert readings on pictures of ambiguous biopsy material [37, 38]. In the context of a SMO, teleconsultations may also be used to combine the convenience and speed of not having to commute to a physician with the security of a personalized expertise [39].

A key strength of this study was that it described a large national cohort of patient-initiated SMOs in France. In contrast with most articles, we described a wide range of diseases encompassing several medical domains. On the other hand, our study also had limitations that deserve to be taken into consideration. Firstly, although there was a countrywide representation of cases, selection bias remained. Secondly, the relatively low number of cases and limited descriptive variables precluded a better analysis of the motivating factors for requesting SMO, or the potential for indication bias. Despite the adjustment applied in the multivariate model, our findings preclude all conclusions regarding the motivations for seeking a SMO. We did not distinguish between diagnostic and therapeutic divergence and could also not ascertain if the recommendation of the SMO were followed. Studies consistently report that only approximately 60 % of patients apply recommendations given in the SMO [14, 40].

The impact of second opinion programs on public health outcomes appears to be minor for some indications, such as for the prevention of caesarean section where 22 cases per 1,000 deliveries were prevented following a SMO program, without a significant impact on patient satisfaction [41]. Other studies have shown that overall satisfaction with SMO programs can be high, irrespective of the presence of a new diagnosis or treatment (95 % satisfaction in one program where treatment was changed in 37 % of cases) [7, 14, 42]. It is worth noting that the participants in our study spontaneously volunteered for a second opinion, and therefore, our results could not be extrapolated at a national level.

## Conclusions

This French nationwide study found a high range of divergent SMO for gynecological, urological, respiratory and endocrine diseases. There is a compelling need for tools to improve care pathways for patients for whom which primary care does not enable satisfactory treatment and produces the need for confirmation of a diagnosis or for more information. Our findings contribute to the ongoing debate on the use of SMOs already initiated for cancer in the French national cancer control plan, with implications for care pathway management and healthcare efficiency strategies. Future research could further explore patient follow-ups after SMOs. Showing a reduction of mortality or hospitalizations would increase the institutional recognition of SMOs and pave the way for a more favorable regulation.

## Supplementary Information

**Additional file 1. **Proportion of divergent diagnoses in the second medical opinion, by disease.

**Additional file 2. **Comparison of patient characteristics according to case complexity.

## Data Availability

All data material can be accessed upon request to the first author Dr Stéphane Sanchez at the following email address stephane.sanchez@hcs-sante.fr.
