# Supplementary material for: Predictive factors of diagnostic and therapeutic divergence in a nationwide cohort of patients seeking second medical opinion
Source: BMC Health Serv Res. 2021 Sep 1;21:902. doi: 10.1186/s12913-021-06936-w (PMC8408960; doi:10.1186/s12913-021-06936-w)
Supplement: Supplementary file 1 — Additional file 1. Proportion of divergent diagnoses in the second medical opinion, by disease. [file 12913_2021_6936_MOESM1_ESM.docx]

**Supplementary Appendix 1.** Proportion of divergent diagnoses in the second medical opinion, by disease.

| **Class of Diseases** | | **Disease specificity** | | **Convergent** | | **Divergent** | | **Percentage divergent** |
| --- | --- | --- | --- | --- | --- | --- | --- | --- |
|  | | Total | | 1050 | | 502 | | 32.3 |
| Orthopedics and rhumatological diseases | | Herniated lumbar disc | | 51 | | 13 | | 20.3 |
| Orthopedics and rhumatological diseases | | Degenerative disc disease | | 31 | | 10 | | 24.4 |
| Orthopedics and rhumatological diseases | | Lumbago (chronic pain) | | 12 | | 10 | | 45.5 |
| Orthopedics and rhumatological diseases | | Lumbar arthritis | | 7 | | 6 | | 46.2 |
| Orthopedics and rhumatological diseases | | Ankle impingement (bone or tissue) | | 5 | | 5 | | 50.0 |
| Orthopedics and rhumatological diseases | | Cervical arthritis | | 3 | | 4 | | 57.1 |
| Orthopedics and rhumatological diseases | | Fracture of humerus (and its consequences) | | 7 | | 4 | | 36.4 |
| Orthopedics and rhumatological diseases | | Fracture of the wrist or forearm (and its consequences) | | 6 | | 4 | | 40.0 |
| Orthopedics and rhumatological diseases | | Meniscal lesions | | 15 | | 4 | | 21.1 |
| Orthopedics and rhumatological diseases | | Arthritis of the hip | | 11 | | 3 | | 21.4 |
| Orthopedics and rhumatological diseases | | Ankle fracture (and its consequences) | | 5 | | 3 | | 37.5 |
| Orthopedics and rhumatological diseases | | Osteoarthritis of the knee | | 7 | | 3 | | 30.0 |
| Orthopedics and rhumatological diseases | | Rotator cuff rupture | | 6 | | 3 | | 33.3 |
| Orthopedics and rhumatological diseases | | Rupture of the anterior cruciate ligament (adults) | | 5 | | 3 | | 37.5 |
| Orthopedics and rhumatological diseases | | Sciatica | | 26 | | 3 | | 10.3 |
| Orthopedics and rhumatological diseases | | Lumbar stenosis (or narrowing of the spinal canal) | | 18 | | 3 | | 14.3 |
| Orthopedics and rhumatological diseases | | Cruralgia | | 2 | | 2 | | 50.0 |
| Orthopedics and rhumatological diseases | | Vertebral frature | | 6 | | 2 | | 25.0 |
| Orthopedics and rhumatological diseases | | Fracture of the foot (and its consequences) | | 5 | | 2 | | 28.6 |
| Orthopedics and rhumatological diseases | | Knee pain | | 1 | | 2 | | 66.7 |
| Orthopedics and rhumatological diseases | | Hallux rigidus | | 1 | | 2 | | 66.7 |
| Orthopedics and rhumatological diseases | | Hallux valgus | | 2 | | 2 | | 50.0 |
| Orthopedics and rhumatological diseases | | Recurrent shoulder dislocation | | 3 | | 2 | | 40.0 |
| Orthopedics and rhumatological diseases | | Pudendal neuralgia | | 1 | | 2 | | 66.7 |
| Orthopedics and rhumatological diseases | | Osteoporosis | | 10 | | 2 | | 16.7 |
| Orthopedics and rhumatological diseases | | Arthritis of the base of the thumb | | 2 | | 2 | | 50.0 |
| Orthopedics and rhumatological diseases | | Ankylosing spondyloarthritis | | 4 | | 2 | | 33.3 |
| Orthopedics and rhumatological diseases | | Carpal tunnel syndrome (and complications) | | 10 | | 2 | | 16.7 |
| Orthopedics and rhumatological diseases | | Tendinitis of the gluteus medius | | 1 | | 2 | | 66.7 |
| Orthopedics and rhumatological diseases | | Wrist trauma / sprain | | 3 | | 2 | | 40.0 |
| Orthopedics and rhumatological diseases | | Plantar fasciitis | | 0 | | 1 | | 100.0 |
| Orthopedics and rhumatological diseases | | Hindfoot arthritis | | 3 | | 1 | | 25.0 |
| Orthopedics and rhumatological diseases | | Camptocormia | | 2 | | 1 | | 33.3 |
| Orthopedics and rhumatological diseases | | Hand surgery | | 8 | | 1 | | 11.1 |
| Orthopedics and rhumatological diseases | | Spine deformation in a child | | 1 | | 1 | | 50.0 |
| Orthopedics and rhumatological diseases | | Deformation of the small toe | | 0 | | 1 | | 100.0 |
| Orthopedics and rhumatological diseases | | Fracture of the shoulder (and its consequences) | | 6 | | 1 | | 14.3 |
| Orthopedics and rhumatological diseases | | Fracture of the hand and/or fingers | | 7 | | 1 | | 12.5 |
| Orthopedics and rhumatological diseases | | Fracture of the elbow (and its consequences) | | 2 | | 1 | | 33.3 |
| Orthopedics and rhumatological diseases | | Ganglion cyst of the write | | 2 | | 1 | | 33.3 |
| Orthopedics and rhumatological diseases | | Lesions of the ankle bones | | 0 | | 1 | | 100.0 |
| Orthopedics and rhumatological diseases | | Kienböck's disease | | 0 | | 1 | | 100.0 |
| Orthopedics and rhumatological diseases | | Paget's disease | | 0 | | 1 | | 100.0 |
| Orthopedics and rhumatological diseases | | osteochondritis dissecans of a femoral condyle (child/adolescent) | | 2 | | 1 | | 33.3 |
| Orthopedics and rhumatological diseases | | Osteoid osteoma | | 3 | | 1 | | 25 |
| Orthopedics and rhumatological diseases | | Neurological pathology of the elbow | | 0 | | 1 | | 100 |
| Orthopedics and rhumatological diseases | | Degenerative flatfoot valgus adult | | 1 | | 1 | | 50 |
| Orthopedics and rhumatological diseases | | Adult scoliosis | | 5 | | 1 | | 16.7 |
| Orthopedics and rhumatological diseases | | Childhood scoliosis | | 7 | | 1 | | 12.5 |
| Orthopedics and rhumatological diseases | | Cervical foraminal stenosis | | 4 | | 1 | | 20 |
| Orthopedics and rhumatological diseases | | Syndactyly (fingers or toes) | | 1 | | 1 | | 50 |
| Orthopedics and rhumatological diseases | | Morton syndrome | | 0 | | 1 | | 100 |
| Orthopedics and rhumatological diseases | | compartment syndrome | | 1 | | 1 | | 50 |
| Orthopedics and rhumatological diseases | | Villonodular synovitis of the ankle | | 0 | | 1 | | 100 |
| Orthopedics and rhumatological diseases | | Heel pain | | 1 | | 1 | | 50 |
| Orthopedics and rhumatological diseases | | Congenital anomaly of the hand in an adult | | 1 | | 0 | | 0 |
| Orthopedics and rhumatological diseases | | Congenital anomaly of the hand in a child | | 1 | | 0 | | 0 |
| Orthopedics and rhumatological diseases | | Congenital anomaly of the foot in a child | | 2 | | 0 | | 0 |
| Orthopedics and rhumatological diseases | | Bucket handle tear of the meniscus | | 4 | | 0 | | 0 |
| Orthopedics and rhumatological diseases | | Arthritis of the shoulder (clavicle-acromion) | | 2 | | 0 | | 0 |
| Orthopedics and rhumatological diseases | | Arthritis of the shoulder (glenohumeral) | | 1 | | 0 | | 0 |
| Orthopedics and rhumatological diseases | | Arthritis of the fingers | | 1 | | 0 | | 0 |
| Orthopedics and rhumatological diseases | | Post-traumatic arthritis of the wrist | | 2 | | 0 | | 0 |
| Orthopedics and rhumatological diseases | | narrowing of the cervical spinal cord | | 6 | | 0 | | 0 |
| Orthopedics and rhumatological diseases | | Femoroacetabular impingement | | 3 | | 0 | | 0 |
| Orthopedics and rhumatological diseases | | Kyphosis | | 1 | | 0 | | 0 |
| Orthopedics and rhumatological diseases | | Torn muscle in the calf | | 3 | | 0 | | 0 |
| Orthopedics and rhumatological diseases | | Chronic rhumatological pain | | 7 | | 0 | | 0 |
| Orthopedics and rhumatological diseases | | Fracture of the head of the humerus in a child | | 1 | | 0 | | 0 |
| Orthopedics and rhumatological diseases | | Fracture of the femur (and its consequences) | | 2 | | 0 | | 0 |
| Orthopedics and rhumatological diseases | | Genu valgum in a child or adolescent | | 2 | | 0 | | 0 |
| Orthopedics and rhumatological diseases | | Dorsal herniated disc | | 8 | | 0 | | 0 |
| Orthopedics and rhumatological diseases | | Instability and laxity - ankle | | 1 | | 0 | | 0 |
| Orthopedics and rhumatological diseases | | Congenital hip dislocation in a child | | 2 | | 0 | | 0 |
| Orthopedics and rhumatological diseases | | Dupuytren's contracture | | 1 | | 0 | | 0 |
| Orthopedics and rhumatological diseases | | Haglund's deformity | | 1 | | 0 | | 0 |
| Orthopedics and rhumatological diseases | | Congenital malformation of the spine | | 2 | | 0 | | 0 |
| Orthopedics and rhumatological diseases | | Dorsal myelopathy | | 1 | | 0 | | 0 |
| Orthopedics and rhumatological diseases | | Osteochondritis of the hip in a child | | 4 | | 0 | | 0 |
| Orthopedics and rhumatological diseases | | Flatfoot valgus in a child | | 1 | | 0 | | 0 |
| Orthopedics and rhumatological diseases | | Painful knee prothesis | | 1 | | 0 | | 0 |
| Orthopedics and rhumatological diseases | | Painful hip prostehsis | | 3 | | 0 | | 0 |
| Orthopedics and rhumatological diseases | | Pseudoarthritis of the shoulder | | 1 | | 0 | | 0 |
| Orthopedics and rhumatological diseases | | Pseudoarthritis of the wrist | | 1 | | 0 | | 0 |
| Orthopedics and rhumatological diseases | | Rhizomelic pseudo-polyarthritis | | 5 | | 0 | | 0 |
| Orthopedics and rhumatological diseases | | Psoriatic arthritis | | 4 | | 0 | | 0 |
| Orthopedics and rhumatological diseases | | Isthmic spondylolisthesis | | 4 | | 0 | | 0 |
| Orthopedics and rhumatological diseases | | Patellofemoral pain syndrome | | 1 | | 0 | | 0 |
| Orthopedics and rhumatological diseases | | Syringomyelia | | 2 | | 0 | | 0 |
| Orthopedics and rhumatological diseases | | Tendinitis and tendon-bursitis of the shoulder | | 3 | | 0 | | 0 |
| Orthopedics and rhumatological diseases | | Tenosynovitis of the long biceps | | 1 | | 0 | | 0 |
| Cardiovascular diseases | | coronary artery disease | | 4 | | 8 | | 66.7 |
| Cardiovascular diseases | | Atrial fibrillation | | 8 | | 3 | | 27.3 |
| Cardiovascular diseases | | Arterial hypertension | | 1 | | 3 | | 75 |
| Cardiovascular diseases | | Phlebitis | | 1 | | 2 | | 66.7 |
| Cardiovascular diseases | | Stenosis of the left anterior descending artery | | 0 | | 2 | | 100 |
| Cardiovascular diseases | | Aneurysm of the thoracic aortia | | 0 | | 1 | | 100 |
| Cardiovascular diseases | | Aneurysm of the popliteal artery | | 0 | | 1 | | 100 |
| Cardiovascular diseases | | Thoracic aortic dissection | | 0 | | 1 | | 100 |
| Cardiovascular diseases | | Mitral valve regurgitation | | 1 | | 1 | | 50 |
| Cardiovascular diseases | | Venous insufficiency | | 0 | | 1 | | 100 |
| Cardiovascular diseases | | Post-phlebitis disease | | 0 | | 1 | | 100 |
| Cardiovascular diseases | | Aortic stenosis | | 2 | | 1 | | 33.3 |
| Cardiovascular diseases | | Brugada syndrome | | 1 | | 1 | | 50 |
| Cardiovascular diseases | | Pelvic congestion syndrome | | 1 | | 1 | | 50 |
| Cardiovascular diseases | | Thoracic outlet syndrome | | 2 | | 1 | | 33.3 |
| Cardiovascular diseases | | Ventricular tachycardia | | 2 | | 1 | | 33.3 |
| Cardiovascular diseases | | Cardiac ablation | | 1 | | 0 | | 0 |
| Cardiovascular diseases | | Aneurysm of the abdominal aortia | | 2 | | 0 | | 0 |
| Cardiovascular diseases | | Mesenteric angina | | 1 | | 0 | | 0 |
| Cardiovascular diseases | | Arteritis of the lower limbs | | 1 | | 0 | | 0 |
| Cardiovascular diseases | | Atherosclerosis of the carotid artery | | 1 | | 0 | | 0 |
| Cardiovascular diseases | | Cardiomyopathy | | 1 | | 0 | | 0 |
| Cardiovascular diseases | | Ventricular extrasystoles | | 1 | | 0 | | 0 |
| Cardiovascular diseases | | Patent foramen ovale | | 1 | | 0 | | 0 |
| Cardiovascular diseases | | Heart failure | | 5 | | 0 | | 0 |
| Cardiovascular diseases | | Bouveret's disease | | 2 | | 0 | | 0 |
| Cardiovascular diseases | | Pericarditis | | 6 | | 0 | | 0 |
| Reproductive diseases | | Female infertility | | 51 | | 16 | | 23.9 |
| Reproductive diseases | | Azoospermia | | 4 | | 4 | | 50 |
| Reproductive diseases | | Oligo-astheno-terato-spermia | | 3 | | 2 | | 40 |
| Reproductive diseases | | Asthenozoospermia | | 0 | | 1 | | 100 |
| Reproductive diseases | | Oligospermia | | 1 | | 1 | | 50 |
| Reproductive diseases | | Teratospermia | | 1 | | 1 | | 50 |
| Reproductive diseases | | Infertility | | 1 | | 0 | | 0 |
| Reproductive diseases | | Fertility preservation | | 1 | | 0 | | 0 |
| Nervous system and sensory organ diseases | | Adult epilepsy | | 4 | | 6 | | 60 |
| Nervous system and sensory organ diseases | | Cervical herniated disc | | 18 | | 5 | | 21.7 |
| Nervous system and sensory organ diseases | | Multiple sclerosis | | 6 | | 5 | | 45.5 |
| Nervous system and sensory organ diseases | | Vascular algia of the face | | 0 | | 3 | | 100 |
| Nervous system and sensory organ diseases | | Childhood epilepsy | | 6 | | 3 | | 33.3 |
| Nervous system and sensory organ diseases | | Parkinson's disease | | 3 | | 2 | | 40 |
| Nervous system and sensory organ diseases | | Sudden onset deafness/loss of hearing in an adult | | 5 | | 2 | | 28.6 |
| Nervous system and sensory organ diseases | | Amblyopia | | 0 | | 1 | | 100 |
| Nervous system and sensory organ diseases | | Cancer of the middle ear | | 1 | | 1 | | 50 |
| Nervous system and sensory organ diseases | | Cataract | | 1 | | 1 | | 50 |
| Nervous system and sensory organ diseases | | Chronic hydrocephalus | | 3 | | 1 | | 25 |
| Nervous system and sensory organ diseases | | Keratoconus | | 0 | | 1 | | 100 |
| Nervous system and sensory organ diseases | | Colloid cyst of the third ventricle | | 5 | | 1 | | 16.7 |
| Nervous system and sensory organ diseases | | Epileptogenic cerebral lesions | | 0 | | 1 | | 100 |
| Nervous system and sensory organ diseases | | Essential tremor | | 1 | | 1 | | 50 |
| Nervous system and sensory organ diseases | | Chiari malformation | | 2 | | 1 | | 33.3 |
| Nervous system and sensory organ diseases | | Meningo-encephalitis | | 0 | | 1 | | 100 |
| Nervous system and sensory organ diseases | | Myopia | | 0 | | 1 | | 100 |
| Nervous system and sensory organ diseases | | Occlusion of the retinal vein | | 1 | | 1 | | 50 |
| Nervous system and sensory organ diseases | | Otospongiosis | | 1 | | 1 | | 50 |
| Nervous system and sensory organ diseases | | Chronic rhino-sinusitis | | 0 | | 1 | | 100 |
| Nervous system and sensory organ diseases | | Strabismus | | 0 | | 1 | | 100 |
| Nervous system and sensory organ diseases | | Transient ischemic attach | | 1 | | 0 | | 0 |
| Nervous system and sensory organ diseases | | Tinnitus | | 1 | | 0 | | 0 |
| Nervous system and sensory organ diseases | | Tonsilectomy | | 2 | | 0 | | 0 |
| Nervous system and sensory organ diseases | | Unruptured intracranial aneurysm | | 4 | | 0 | | 0 |
| Nervous system and sensory organ diseases | | Cavernoma | | 4 | | 0 | | 0 |
| Nervous system and sensory organ diseases | | Craniostenosis | | 1 | | 0 | | 0 |
| Nervous system and sensory organ diseases | | Detached retina | | 2 | | 0 | | 0 |
| Nervous system and sensory organ diseases | | Age-related macular degeneration | | 2 | | 0 | | 0 |
| Nervous system and sensory organ diseases | | Dystonia | | 1 | | 0 | | 0 |
| Nervous system and sensory organ diseases | | Image result for corneal ectasia | | 1 | | 0 | | 0 |
| Nervous system and sensory organ diseases | | Corneal Ectasia | | 8 | | 0 | | 0 |
| Nervous system and sensory organ diseases | | Subdural hematoma in a child | | 1 | | 0 | | 0 |
| Nervous system and sensory organ diseases | | Chronic subdural hematoma | | 2 | | 0 | | 0 |
| Nervous system and sensory organ diseases | | Corneal infection with contact lenses | | 1 | | 0 | | 0 |
| Nervous system and sensory organ diseases | | Non-ruptured intracranial arteriovenous malformation | | 3 | | 0 | | 0 |
| Nervous system and sensory organ diseases | | Epiretinal membrane | | 3 | | 0 | | 0 |
| Nervous system and sensory organ diseases | | Cerebral metastasis | | 2 | | 0 | | 0 |
| Nervous system and sensory organ diseases | | Vestibular schwannoma | | 4 | | 0 | | 0 |
| Nervous system and sensory organ diseases | | Trigeminal neuralgia | | 2 | | 0 | | 0 |
| Nervous system and sensory organ diseases | | Multiple sclerosis (old) | | 1 | | 0 | | 0 |
| Nervous system and sensory organ diseases | | Deafness (child) | | 1 | | 0 | | 0 |
| Nervous system and sensory organ diseases | | Cubital tunnel syndrome | | 1 | | 0 | | 0 |
| Nervous system and sensory organ diseases | | Vertigo | | 1 | | 0 | | 0 |
| Skin disorders | | Dermatosis | | 3 | | 3 | | 50 |
| Skin disorders | | Keloid acne | | 1 | | 1 | | 50 |
| Skin disorders | | Cutaneous carcinoma | | 2 | | 1 | | 33.3 |
| Skin disorders | | Palmar and/or axillary hyperhidrosis | | 0 | | 1 | | 100 |
| Skin disorders | | Androgenetic alopecia | | 1 | | 0 | | 0 |
| Skin disorders | | Pediatric dermatology | | 1 | | 0 | | 0 |
| Skin disorders | | Cyst on the scalp | | 1 | | 0 | | 0 |
| Skin disorders | | Lichen planus | | 1 | | 0 | | 0 |
| Skin disorders | | Lichen sclerosus | | 1 | | 0 | | 0 |
| Skin disorders | | Lupus | | 2 | | 0 | | 0 |
| Skin disorders | | Morphea | | 3 | | 0 | | 0 |
| Skin disorders | | Psoriasis in the hair | | 1 | | 0 | | 0 |
| Digestive system diseases | | Crohn's disease | | 3 | | 5 | | 62.5 |
| Digestive system diseases | | Pancreatitis (acute. chronic) | | 1 | | 3 | | 75 |
| Digestive system diseases | | Diverticulosis | | 3 | | 2 | | 40 |
| Digestive system diseases | | Hepatic adenoma | | 1 | | 1 | | 50 |
| Digestive system diseases | | Functional diarrhea | | 0 | | 1 | | 100 |
| Digestive system diseases | | Gallstones | | 3 | | 1 | | 25 |
| Digestive system diseases | | Crohn's disease (old) | | 0 | | 1 | | 100 |
| Digestive system diseases | | Rectocele | | 1 | | 1 | | 50 |
| Digestive system diseases | | Ulcerative colitis and other unspecified colitis | | 1 | | 1 | | 50 |
| Digestive system diseases | | Ampullary carcinoma | | 1 | | 0 | | 0 |
| Digestive system diseases | | Bariatric surgery | | 1 | | 0 | | 0 |
| Digestive system diseases | | Bile duct cancer | | 1 | | 0 | | 0 |
| Digestive system diseases | | Cirrhosis of the liver | | 2 | | 0 | | 0 |
| Digestive system diseases | | Functional dyspepsia | | 1 | | 0 | | 0 |
| Digestive system diseases | | Hemorrhoids | | 2 | | 0 | | 0 |
| Digestive system diseases | | Auto-immune hepatitis | | 4 | | 0 | | 0 |
| Digestive system diseases | | Hepatitis B | | 1 | | 0 | | 0 |
| Digestive system diseases | | Inguinal hernia | | 1 | | 0 | | 0 |
| Digestive system diseases | | Umbilical hernia | | 1 | | 0 | | 0 |
| Digestive system diseases | | Hyperbilirubinemia | | 1 | | 0 | | 0 |
| Digestive system diseases | | Renal insufficiency | | 2 | | 0 | | 0 |
| Digestive system diseases | | Coeliac disease | | 1 | | 0 | | 0 |
| Digestive system diseases | | Gastro-oesophageal reflux | | 1 | | 0 | | 0 |
| Tumours, cancer, hematological diseases | Prostate cancer | | 30 | | 16 | | 34.8 | |
| Tumours, cancer, hematological diseases | Bladder cancer | | 8 | | 4 | | 33.3 | |
| Tumours, cancer, hematological diseases | Thyroid cancer | | 2 | | 3 | | 60 | |
| Tumours, cancer, hematological diseases | Salivary gland cancer | | 1 | | 2 | | 66.7 | |
| Tumours, cancer, hematological diseases | Pancreatic cancer | | 6 | | 2 | | 25 | |
| Tumours, cancer, hematological diseases | Kidney cancer | | 5 | | 2 | | 28.6 | |
| Tumours, cancer, hematological diseases | Breast cancer | | 26 | | 2 | | 7.1 | |
| Tumours, cancer, hematological diseases | Meningioma | | 16 | | 2 | | 11.1 | |
| Tumours, cancer, hematological diseases | Ovarian cancer | | 9 | | 1 | | 10 | |
| Tumours, cancer, hematological diseases | Cancer of the anal canal | | 1 | | 1 | | 50 | |
| Tumours, cancer, hematological diseases | Cervical cancer | | 3 | | 1 | | 25 | |
| Tumours, cancer, hematological diseases | Colon cancer | | 5 | | 1 | | 16,7 | |
| Tumours, cancer, hematological diseases | Cancer of the larynx | | 1 | | 1 | | 50 | |
| Tumours, cancer, hematological diseases | Cancer of the nose, sinus, nasal cavities | | 0 | | 1 | | 100 | |
| Tumours, cancer, hematological diseases | Rectal cancer | | 4 | | 1 | | 20 | |
| Tumours, cancer, hematological diseases | Testicular cancer | | 2 | | 1 | | 33.3 | |
| Tumours, cancer, hematological diseases | Hemochromatosis | | 2 | | 1 | | 33,3 | |
| Tumours, cancer, hematological diseases | Lymphoma | | 8 | | 1 | | 11,1 | |
| Tumours, cancer, hematological diseases | Myelofibrosis | | 1 | | 1 | | 50 | |
| Tumours, cancer, hematological diseases | Thrombocytemia | | 1 | | 1 | | 50 | |
| Tumours, cancer, hematological diseases | Cerebral tumour | | 9 | | 1 | | 10 | |
| Tumours, cancer, hematological diseases | Intraductal papillary mucinous neoplasm of the pancreas | | 3 | | 1 | | 25 | |
| Tumours, cancer, hematological diseases | Neuroendocrine tumour of the pancreas | | 0 | | 1 | | 100 | |
| Tumours, cancer, hematological diseases | Stomach cancer | | 2 | | 0 | | 0 | |
| Tumours, cancer, hematological diseases | Cancer of the tonsils or other oropharyngeal site (base of the tongue, palate) | | 1 | | 0 | | 0 | |
| Tumours, cancer, hematological diseases | Cancer of the biliary ducts | | 2 | | 0 | | 0 | |
| Tumours, cancer, hematological diseases | Cancer of the cardia | | 1 | | 0 | | 0 | |
| Tumours, cancer, hematological diseases | Uterine cancer | | 4 | | 0 | | 0 | |
| Tumours, cancer, hematological diseases | Duodenal cancer | | 1 | | 0 | | 0 | |
| Tumours, cancer, hematological diseases | Nasopharyngeal cancer | | 1 | | 0 | | 0 | |
| Tumours, cancer, hematological diseases | Cancer of the peritoneum | | 2 | | 0 | | 0 | |
| Tumours, cancer, hematological diseases | Lung cancer (adenocarcinoma) | | 4 | | 0 | | 0 | |
| Tumours, cancer, hematological diseases | Lung cancer (large-cell, undifferentiated) | | 1 | | 0 | | 0 | |
| Tumours, cancer, hematological diseases | Lung cancer (Squamous cell carcinoma) | | 3 | | 0 | | 0 | |
| Tumours, cancer, hematological diseases | Lung cancer (small-cell, neuroendocrine tumour) | | 1 | | 0 | | 0 | |
| Tumours, cancer, hematological diseases | Metastatic liver cancer | | 5 | | 0 | | 0 | |
| Tumours, cancer, hematological diseases | Primary liver cancer | | 1 | | 0 | | 0 | |
| Tumours, cancer, hematological diseases | Acute leukemia | | 1 | | 0 | | 0 | |
| Tumours, cancer, hematological diseases | Acute myeloblastic leukemia in an adult | | 1 | | 0 | | 0 | |
| Tumours, cancer, hematological diseases | Primary Polycythemia (Vaquez disease) | | 1 | | 0 | | 0 | |
| Tumours, cancer, hematological diseases | Melanoma | | 5 | | 0 | | 0 | |
| Tumours, cancer, hematological diseases | Myelodysplasia | | 1 | | 0 | | 0 | |
| Tumours, cancer, hematological diseases | Cutaneous sarcoma | | 1 | | 0 | | 0 | |
| Tumours, cancer, hematological diseases | Soft-tissue sarcoma | | 1 | | 0 | | 0 | |
| Tumours, cancer, hematological diseases | Intradural extramedullary tumour | | 1 | | 0 | | 0 | |
| Tumours, cancer, hematological diseases | Neuroendocrine tumour of the large intestine | | 2 | | 0 | | 0 | |
| Tumours, cancer, hematological diseases | Neuroendocrine tumour of the colon | | 1 | | 0 | | 0 | |
| Endocrine and metabolic diseases | Goiter or thyroid nodule | | 7 | | 5 | | 41.7 | |
| Endocrine and metabolic diseases | Hyperthyroidism | | 10 | | 5 | | 33.3 | |
| Endocrine and metabolic diseases | Hypothyroidism | | 3 | | 3 | | 50 | |
| Endocrine and metabolic diseases | Type 2 diabetes mellitus | | 4 | | 2 | | 33.3 | |
| Endocrine and metabolic diseases | Grave's disease | | 2 | | 2 | | 50 | |
| Endocrine and metabolic diseases | Pituitary adenoma | | 3 | | 1 | | 25 | |
| Endocrine and metabolic diseases | Hypothyroidism (old) | | 0 | | 1 | | 100 | |
| Endocrine and metabolic diseases | Primary adrenal insufficiency (Addison's disease) | | 0 | | 1 | | 100 | |
| Endocrine and metabolic diseases | Acromegaly | | 1 | | 0 | | 0 | |
| Endocrine and metabolic diseases | Hyperparathyroidism | | 1 | | 0 | | 0 | |
| Endocrine and metabolic diseases | Pituitary insufficiency in a child | | 1 | | 0 | | 0 | |
| Endocrine and metabolic diseases | Antiphospholipid syndrome | | 2 | | 0 | | 0 | |
| Endocrine and metabolic diseases | Hashimoto's disease | | 4 | | 0 | | 0 | |
| Gynecological diseases | Endometriosis | | 90 | | 137 | | 60.4 | |
| Gynecological diseases | Uterine fibrosis | | 12 | | 11 | | 47.8 | |
| Gynecological diseases | HPV and anal-genital lesions | | 7 | | 1 | | 12.5 | |
| Gynecological diseases | Genital prolapse | | 2 | | 1 | | 33.3 | |
| Gynecological diseases | Fibroadenoma | | 1 | | 0 | | 0 | |
| Gynecological diseases | Ovarian cysts | | 9 | | 0 | | 0 | |
| Respiratory diseases | Chronic obstructive pulmonary disease | | 3 | | 4 | | 57.1 | |
| Respiratory diseases | Sphenoid sinusitis | | 6 | | 2 | | 25 | |
| Respiratory diseases | Pulmonary emphysema | | 1 | | 1 | | 50 | |
| Respiratory diseases | Idiopathic pulmonary fibrosis | | 0 | | 1 | | 100 | |
| Respiratory diseases | Chronic cough | | 0 | | 1 | | 100 | |
| Urological diseases | Prostate adenoma | | 14 | | 14 | | 50,0 | |
| Urological diseases | | Neurogenic bladder | | 0 | | 3 | | 100 |
| Urological diseases | | Urinary tract infection (recurrent) | | 3 | | 2 | | 40 |
| Urological diseases | | Urinary lithiasis | | 3 | | 2 | | 40 |
| Urological diseases | | Urinary incontinence | | 0 | | 1 | | 100 |
| Urological diseases | | Neuromyelitis optica | | 1 | | 0 | | 0 |
| Other | | Lyme disease | | 1 | | 2 | | 66.7 |
| Other | | Renal vasculitis | | 1 | | 0 | | 0 |
