# Supplementary material for: Predictive factors of diagnostic and therapeutic divergence in a nationwide cohort of patients seeking second medical opinion
Source: BMC Health Serv Res. 2021 Sep 1;21:902. doi: 10.1186/s12913-021-06936-w (PMC8408960; doi:10.1186/s12913-021-06936-w)
Supplement: Supplementary file 2 — Additional file 2. Comparison of patient characteristics according to case complexity. [file 12913_2021_6936_MOESM2_ESM.docx]

**Supplementary Appendix 2.** Comparison of patient characteristics according to case complexity

| **Variable** | **Simple** | **Complex** | **Missing data** | **P-value** |
| --- | --- | --- | --- | --- |
| n | 473 | 191 | 25.6 |  |
| **Satisfaction, n (%)** |  |  | 0.3 | 0.41 |
| *Moderately satisfied* | 6 (1.3) | 2 (1.1) |  |  |
| *Satisfied* | 74 (15.7) | 38 (20.0) |  |  |
| *Very satisfied* | 391 (83.0) | 150 (78.9) |  |  |
| Expert Recommendation, n (%) | 471 (99.6) | 189 (99.0) | 2.2 | 0.32 |
| Expert choice= Patient, n (%) | 327 (69.1) | 140 (73.3) | 0.0 | 0.33 |
| Time - median [IQR] | 1.79 [0.38, 4.27] | 2.67 [0.91, 5.18] | 0.3 | 0.01 |
| Male sex, n (%) | 197 (41.7) | 66 (34.7) | 0.2 | 0.11 |
| GP aware of request for second opinion, n (%) | 223 (47.9) | 88 (46.6) | 1.7 | 0.83 |
| **Employment status, n (%)** |  |  | 30.2 | 0.85 |
| *Self-employed, business owners, tradesperson* | 16 (4.4) | 4 (2.8) |  |  |
| *Other* | 39 (10.8) | 19 (13.3) |  |  |
| *Currently unemployed* | 23 (6.4) | 11 (7.7) |  |  |
| *Upper level management* | 80 (22.2) | 30 (21.0) |  |  |
| *Employee* | 109 (30.2) | 49 (34.3) |  |  |
| *Manual labourer* | 10 (2.8) | 4 (2.8) |  |  |
| *Intermediate profession* | 15 (4.2) | 5 (3.5) |  |  |
| *Retired* | 69 (19.1) | 21 (14.7) |  |  |
| **Class of diseases, n (%)** |  |  | 0.20 | 0.053 |
| *Cardiovascular diseases* | 22 (4.7) | 7 (3.7) |  |  |
| *Reproductive diseases* | 27 (5.7) | 3 (1.6) |  |  |
| *Nervous system/sensory organ diseases* | 31 (6.6) | 25 (13.1) |  |  |
| *Skin disorders* | 7 (1.5) | 3 (1.6) |  |  |
| *Digestive system diseases* | 15 (3.2) | 6 (3.1) |  |  |
| *Tumours, cancer, hematology* | 75 (15.9) | 30 (15.7) |  |  |
| *Endocrine/metabolic diseases* | 15 (3.2) | 12 (6.3) |  |  |
| *Gynecological diseases* | 82 (17.4) | 37 (19.4) |  |  |
| *Respiratory diseases* | 7 (1.5) | 2 (1.0) |  |  |
| *Orthopedics/rhumatological diseases* | 179 (38.0) | 64 (33.5) |  |  |
| *Urological diseases* | 11 (2.3) | 2 (1.0) |  |  |
| **Age category, n (%)** |  |  | 0.0 | 0.07 |
| *Children<18 years* | 22 (4.7) | 6 (3.1) |  |  |
| *Adult aged 19 to 35 years* | 104 (22.0) | 55 (28.8) |  |  |
| *Adult aged 36 to 50 years* | 132 (27.9) | 64 (33.5) |  |  |
| *Adult aged 51 to 65 years* | 124 (26.2) | 40 (20.9) |  |  |
| *Adult aged 66 to 80 years* | 86 (18.2) | 23 (12.0) |  |  |
| *Adult aged 81 to 95 years* | 5 (1.1) | 3 (1.6) |  |  |
| **Region of residence, n (%)** |  |  | 9.4 | 0.93 |
| *Auvergne Rhône-Alpes, Bourgogne, Franche-Comté* | 53 (12.2) | 22 (12.7) |  |  |
| *Bretagne, Normandie, Pays de la Loire, Centre-Val de Loire* | 65 (15.0) | 20 (11.6) |  |  |
| *Overseas territories* | 5 (1.2) | 1 (0.6) |  |  |
| *Corsica* | 1 (0.2) | 2 (1.2) |  |  |
| *Hauts-de-France, Grand-Est* | 89 (20.5) | 34 (19.7) |  |  |
| *Ile-de-France* | 120 (27.6) | 44 (25.4) |  |  |
| *Nouvelle-Aquitaine* | 29 (6.7) | 12 (6.9) |  |  |
| *PACA, Occitanie* | 72 (16.6) | 38 (22.0) |  |  |
